# An overview of topic modeling and its current applications in bioinformatics

**DOI:** 10.1186/s40064-016-3252-8

**Published:** 2016-09-20

**Authors:** Lin Liu, Lin Tang, Wen Dong, Shaowen Yao, Wei Zhou

**Affiliations:** 1 School of Information, Yunnan University, Kunming, 650091 Yunnan China; 2 School of Information (Key Laboratory of Educational Informatization for Nationalities Ministry of Education), Yunnan Normal University, Kunming, 650092 Yunnan China; 3 Key Laboratory of Educational Informatization for Nationalities Ministry of Education, Yunnan Normal University, Kunming, 650092 Yunnan China; 4 National Pilot School of Software, Yunnan University, Kunming, 650091 Yunnan China

**Keywords:** Topic model, Bioinformatics, Probabilistic generative model, Clustering, Classification

## Abstract

**Background:**

With the rapid accumulation of biological datasets, machine learning methods designed to automate data analysis are urgently needed. In recent years, so-called topic models that originated from the field of natural language processing have been receiving much attention in bioinformatics because of their interpretability. Our aim was to review the application and development of topic models for bioinformatics.

**Description:**

This paper starts with the description of a topic model, with a focus on the understanding of topic modeling. A general outline is provided on how to build an application in a topic model and how to develop a topic model. Meanwhile, the literature on application of topic models to biological data was searched and analyzed in depth. According to the types of models and the analogy between the concept of document-topic-word and a biological object (as well as the tasks of a topic model), we categorized the related studies and provided an outlook on the use of topic models for the development of bioinformatics applications.

**Conclusion:**

Topic modeling is a useful method (in contrast to the traditional means of data reduction in bioinformatics) and enhances researchers’ ability to interpret biological information. Nevertheless, due to the lack of topic models optimized for specific biological data, the studies on topic modeling in biological data still have a long and challenging road ahead. We believe that topic models are a promising method for various applications in bioinformatics research.

## Background

A topic model is a kind of a probabilistic generative model that has been used widely in the field of computer science with a specific focus on text mining and information retrieval in recent years. Since this model was first proposed, it has received a lot of attention and gained widespread interest among researchers in many research fields. So far, besides text mining, there also have been successful applications in the fields of computer vision (Fei–Fei and Perona 2005; Luo et al. 2015), population genetics, and social networks (Jiang et al. 2015).

The origin of a topic model is latent semantic indexing (LSI) (Deerwester et al. 1990); it has served as the basis for the development of a topic model. Nevertheless, LSI is not a probabilistic model; therefore, it is not an authentic topic model. Based on LSI, probabilistic latent semantic analysis (PLSA) (Hofmann 2001) was proposed by Hofmann and is a genuine topic model. Published after PLSA, latent Dirichlet allocation (LDA) proposed by Blei et al. (2003) is an even more complete probabilistic generative model and is the extension of PLSA. Nowadays, there is a growing number of probabilistic models that are based on LDA via combination with particular tasks. Nonetheless, all the above-mentioned topic models have initially been introduced in the text analysis community for unsupervised topic discovery in a corpus of documents.

Since the emergence of topic models, researchers have introduced this approach into the fields of biological and medical document mining. Because of its superiority in analysis of large-scale document collections, better results have been obtained in such fields as biological/biomedical text mining (Andrzejewski 2006; Wang et al. 2009, 2013, 2016; Bisgin et al. 2011, 2012; Chen et al. 2012c; Song and Kim 2013) and clinical informatics (Arnold et al. 2010; Sarioglu et al. 2012; Zeng et al. 2012; Zhang et al. 2012b; Howes et al. 2013; Sarioglu et al. 2013; Hu et al. 2014; Huang et al. 2014). On the other hand, most of these studies follow the classic text-mining method of a topic model.

In recent years, we have been witnessing exponential growth of biological data, such as microarray datasets. This situation also poses a great challenge, namely, how to extract hidden knowledge and relations from these data. As mentioned above, topic models have emerged as an effective method for discovering useful structure in collections. Therefore, a growing number of researchers are beginning to integrate topic models into various biological data, not only document collections. In these studies, we find that topic models act as more than a classification or clustering approach. They can model a biological object in terms of hidden “topics” that can reflect the underlying biological meaning more comprehensively. Therefore, topic models were recently shown to be a powerful tool for bioinformatics. In this paper, the existing studies on topic modeling in biological data are analyzed from different points of view, and then the problems and prospects are discussed. To the best of our knowledge, this is the first effort to review the application and development of topic models for bioinformatics. In contrast, the studies related to topic models applied to pure biological or medical text mining are outside the scope of this paper.

The rest of this paper is structured as follows. In “Topic modeling” section, the general outline of how to build an application in accordance with a topic model is given. In particular, LDA and PLSA are presented by means of the terminology and notation of the document analysis context. “The development of a topic model” and “The toolkits for topic models” sections summarize a large number of topic models that evolved from LDA and the existing topic model toolkits. In “The use of topic models in bioinformatics” sections, numerous relevant papers on topic models—as applied to bioinformatics—are discussed in keeping with three themes: the tasks of a topic model, the types of models, and an analogy between the concept “document-topic-word” and a biological object. In “The trends in applications of topic models to bioinformatics” sections, we give our thoughts on some of the promising unexplored directions for the use of topic modeling in biological applications. Finally, the conclusions are drawn.

## Topic modeling

**Fig. 1:**
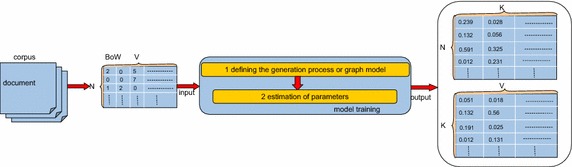
The diagram of topic modeling

To better understand how to use a topic model in bioinformatics, we first describe the basic ideas behind topic modeling by means of a diagram. Figure 1 (The diagram of topic modeling) illustrates the key steps of topic modeling, including the bag of words (BoW), model training, and model output. We first assume that there are *N* documents, *V* words, and *K* topics in a corpus. Then, we discuss each component of this diagram in detail.

### The BoW

In natural language processing, a document is usually represented by a BoW that is actually a word-document matrix. An example of a BoW is shown in Table 1.

**Table 1:** An example of a BoW

	d_1_	d_2_	d_3_	d_4_	d_5_	d_6_
Gene	2	0	3	0	0	0
Protein	0	5	0	0	0	0
Pathway	1	2	0	0	0	0
Microarray	0	0	3	6	0	0

As shown in Table 1, there are four words (gene, protein, pathway, and microarray) and six documents (*d*_1_–*d*_6_) in this corpus. Value *w*_*ij*_ in the matrix represents the frequency of word *i* in document *j*. For example, *w*_3,1_ = 1 means that the frequency of the word “pathway” in document *d*_1_ is 1.0. It is obvious that the number of words is fixed in a corpus, and the collection of these words constitutes a vocabulary. In short, the corpus is represented by the BoW it contains. A BoW is a simplified representation of a corpus as the input of topic modeling. Likewise, if we want to process biological data rather than a corpus, we also need to represent biological data as a BoW: to specify which is the document and which is the word in the field of biology. For instance, in the problem of genomic sequence classification, La Rosa et al. (2015) consider genomic sequences to be documents and small fragments of a DNA string of size *k* to be words. Then, the BoW of genomic sequences can be calculated easily. After construction of the BoW, it serves as the input of the next step in topic modeling. Suppose there are *N* documents and *V* words in a corpus; thus, the BoW of this corpus is an *N* × *V* matrix.

From the description of the BoW above, we can deduce that the order of words in a document does not affect the representation of the BoW. Put another way, the words in the document are exchangeable. Moreover, the documents in a corpus are independent: there is no relation among the documents. The exchangeability of words and documents could be called the basic assumptions of a topic model. These assumptions are available in both PLSA and LDA. Nevertheless, in several variants of topic models, a basic assumption was relaxed. The summary of variants of LDA is provided in section “The development of a topic model”.

### Model training

In a BoW, the dimensionality of word space may be enormous, and the BoW reflects only the words of the original texts. In contrast, the most important thing people expect to know about a document is the themes rather than words. The aim of topic modeling is to discover the themes that run through a corpus by analyzing the words of the original texts. We call these themes “topics.” The classic topic models are unsupervised algorithms (that do not require any prior annotations or labeling of the documents), and the “topics” were discovered during model training.

#### The definition of a topic

**Fig. 2:**
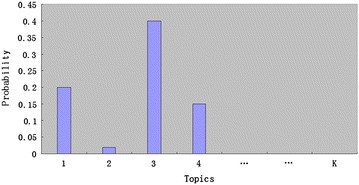
The topic distribution of a document

In topic modeling, a “topic” is viewed as a probability distribution over a fixed vocabulary. As an example, Table 2 (The top five most frequent words from three topics) illustrates three “topics” that were discovered in a corpus, including “Protein,” “Cancer,” and “Computation” (Blei 2012). As shown in Table 2, the probabilities of each word in a “topic” were sorted in the descending order. The top five most frequent words reflect the related concepts of each “topic”: “Topic 1” is about a protein, “Topic 2” is about cancer, and “Topic 3” is about computation. In short, each “topic” is a mixture of “words” in a vocabulary. Similarly, in topic modeling, each document is a mixture of “topics.” As shown in Fig. 2 (The topic distribution of a document), we assumed that *K* is the number of topics.

**Table 2:** The top five most frequent words from three topics

Topics	Protein	Cancer	Computation
Words	Protein	Tumor	Computer
	Cell	Cancer	Model
	Gene	Diseases	Algorithm
	DNA	Death	Data
	Polypeptide	Medical	Mathematical

Above all, the key idea behind topic modeling is that documents show multiple topics, and therefore the key question of topic modeling is how to discover a topic distribution over each document and a word distribution over each topic, which represent an *N* × *K* matrix and a *K* × *V* matrix, respectively. The output of a topic model is then obtained in the next two steps.

#### The generative process

First, topic modeling needs to simulate the generative process of documents. Each document is assumed to be generated as follows: for each word in this document, choose a topic assignment and choose the word from the corresponding topic. PLSA and LDA are relatively simple topic models; in particular, other topic modes that appeared in recent years are more or less related to LDA. Therefore, understanding LDA is important for the extended application of topic models. We use PLSA and LDA as examples to describe the generative process in this paper.

**Fig. 3:**
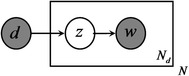
The graphical model of PLSA

In PLSA, suppose *d* denotes the label of a document, *z* is a topic, *w* represents a word, and *N*_*d*_ is the number of words in document *d*. Therefore, *P*(*z*|*d*) denotes the probability of topic *z* in document *d*, and *P*(*w*|*z*) means the probability of word *w* in topic *z*. Then, for PLSA, the generative procedure for each word in the document is as follows: (a) Randomly choose a topic from the distribution over topics (*P*(*z*|*d*)); (b) randomly choose a word from the corresponding distribution over the vocabulary (*P*(*w*|*z*)). The pseudo-code is as follows:
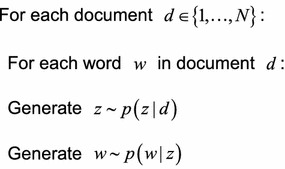


Besides the descriptive approach of the generative process above, a graphical model can also reflect the generative process of documents. As shown in Fig. 3 (The graphical model of PLSA), the box indicates repeated contents; the number in the lower right corner is the number of repetitions. The gray nodes represent observations; white nodes represent hidden random variables or parameters. The arrows denote dependences.

In LDA, the two probability distributions, *p*(*z*|*d*) and *p*(*w*|*z*), are assumed to be multinomial distributions. Thus, the topic distributions in all documents share the common Dirichlet prior ${\varvec{\upalpha}}$, and the word distributions of topics share the common Dirichlet prior ${\varvec{\upeta}}$. Given the parameters ${\varvec{\upalpha}}$ and ${\varvec{\upeta}}$ for document *d*, parameter *θ*_*d*_ of a multinomial distribution over *K* topics is constructed from Dirichlet distribution *Dir*(*θ*_*d*_|*α*). Similarly, for topic *k*, parameter *β*_*k*_ of a multinomial distribution over *V* words is derived from Dirichlet distribution *Dir*(*β*_*k*_|*η*). As a conjugate prior for the multinomial, the Dirichlet distribution is a convenient choice as a prior and can simplify the statistical inference in LDA. Therefore, in PLSA, by contrast, any common prior probability distribution was not specified for *p*(*z*|*d*) and*p*(*w*|*z*). Naturally, there are no ${\varvec{\upalpha}}$ and ${\varvec{\upeta}}$ in the generative process of PLSA.

**Fig. 4:**
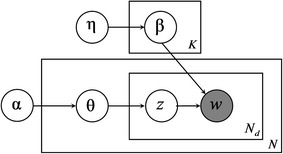
The graphical model of LDA

Then, we can summarize LDA as a generative procedure:
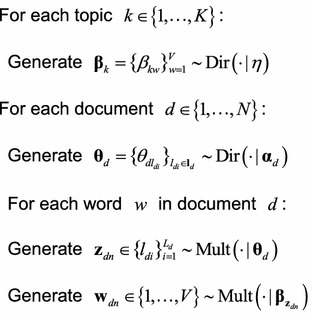


Likewise, we can use a graphical model to represent LDA, as shown in Fig. 4 (The graphical model of LDA).

#### The parameter estimation

As described above, the goal of topic modeling is to automatically discover the topics in a collection of documents. The documents themselves are examined, whereas the topic structure—the topics, per-document topic distributions, and the per-document per-word topic assignments—is hidden structure. The central computational problem for topic modeling is how to use the documents under study to infer the hidden topic structure. This task can be thought of as a “reversal” of the generative process; the task of parameter estimation can be summarized as follows: given the corpus, estimate the posterior distribution of unknown model parameters and hidden variables.

According to the generative procedure of PLSA, the log-likelihood of a corpus is given by

$$ L = \sum\limits_{d \in N} {\sum\limits_{w \in V} {n\left( {d,w} \right)} } \log p\left( {d,w} \right) $$

where *n*(*d*, *w*) denotes the number of times word *w* appeared in document *d*, and log *p*(*d*, *w*) means the probability of (*d*, *w*). Then, the maximum likelihood estimator is used to obtain the model parameters (*p*(*z*|*d*), *p*(*w*|*z*)), such as the expectation maximization algorithm (EM) (Moon 1996).

For an LDA model, given the parameters ${\varvec{\upalpha}}$ and ${\varvec{\upeta}}$, the empirical values are ${\varvec{\upalpha}} = 50/K$ and ${\varvec{\upeta}} = 0.01$. The joint distribution of topic mixture ${\varvec{\uptheta}}$, word mixture ${\varvec{\upbeta}}$, a set of *K* topics *z*, and a set of *N* words *w* that constitute the document is expressed as

$$ p\left( {{\varvec{\upbeta}},{\varvec{\uptheta}},w,z|{\mathbf{\alpha ,\eta }}} \right) = \prod\limits_{d = 1}^{N} {p\left( {{\varvec{\uptheta}}_{d} |{\varvec{\upalpha}}} \right)} \prod\limits_{n = 1}^{{N_{d} }} {p\left( {{\mathbf{z}}_{dn} |{\varvec{\uptheta}}_{d} } \right)p\left( {{\mathbf{w}}_{dn} |{\mathbf{z}}_{dn} ,{\varvec{\upbeta}}} \right)} \prod\limits_{k = 1}^{K} {p\left( {{\varvec{\upbeta}}_{k} |{\varvec{\upeta}}} \right)} $$

Via the joint distribution, we can estimate $p\left( {{\varvec{\upbeta}},{\varvec{\uptheta}},z|w} \right)$, the posterior distribution of unknown model parameters and hidden variables: the central task of learning in a topic model. Classic approaches to an inference algorithm in LDA are expectation-propagation (EP) (Minka and Lafferty 2002), collapsed Gibbs sampling (Griffiths and Steyvers 2004), and variational Bayesian inference (VB) (Blei et al. 2003). Besides, Teh et al. (2006b) proposed a collapsed variational Bayesian, which combines collapsed Gibbs sampling and VB. Every kind of algorithm has its own advantages: the variational approach is arguably faster computationally, but the Gibbs sampling approach is in principle more accurate (Porteous et al. 2008). We need to choose them according to efficiency, complexity, accuracy, and the generative process. Regardless of the method that we choose, their aim is the same: given the objective functions for optimization, to obtain an estimate of a parameter.

For model training, the inference algorithm of parameters is based on the generative process or a graph model and is the most complex and important stage in topic modeling. For brevity, however, these methods will not be described in detail. Moreover, if we use only LDA, PLSA, or other existing topic models directly, their inference algorithm of parameters is ready-made, and the tasks that we need to do are construction of data input and parameter initialization.

### Model outputs

For PLSA and LDA, the outputs of the model include two matrices: one is the topic probability distributions over documents, represented by an *N* × *K* matrix; the other is the word probability distributions over topics, represented by a *K* × *V* matrix. “Topics” can be identified by estimating the parameters in the case of known documents. If the number of “topics” was specified as *K*, then *K* “topics” could be obtained through model training. After that, the word term space of documents is transformed into “topic” space. It is obvious that “topic” space is smaller than word space (*K* < *V*), and moreover, examining a document at the topic level instead of at the word level is beneficial for discovery of meaningful structure of the documents.

The output of a topic model actually reflects the ability to cluster for the corpus. This is because documents with a similar topic probability distribution can be grouped together. Nonetheless, a topic model is not only a clustering algorithm. In contrast to other black-box algorithms, a topic model can interpret the clustering results by the word probability distributions over topics. Meanwhile, it allows data to come from a mixture of topics rather than from only one topic. These characteristics may be crucial for various applications.

## The development of a topic model

The simple PLSA or LDA model offers a powerful tool for discovering and exploiting the hidden “topics” in large document collections. We find that, as probabilistic models, the basic topic models such as LDA can be easily modified for a more complicated application. Therefore, since its introduction, LDA has been extended and adapted in many ways. The major extension models of LDA are summarized below.

### A supervised topic model

As an unsupervised learning model, LDA can discover underlying topics in unlabeled data. Nevertheless, “topics” discovered in an unsupervised way may not match the true topics in the data. Therefore, many researchers modified LDA in a supervised learning manner, which can introduce known label information into the topic discovery process.

The typical supervised topic models include supervised LDA (sLDA) (Mcauliffe and Blei 2008), the discriminative variation on LDA (discLDA) (Lacoste-Julien et al. 2009), and maximum entropy discrimination LDA (medLDA) (Zhu et al. 2012). For example, sLDA associates each document with an observable continuous response variable, and models the response variables using normal linear regression. A multilabel topic model called labeled LDA (LLDA) (Ramage et al. 2009) extends previous supervised models to allow for multiple labels of documents, and the relation of labels to topics represents one-to-one mapping. Partially labeled LDA (PLLDA) (Ramage et al. 2011) further extends LLDA to have latent topics not present in the document labels.

### Extension of topic attributes

In an LDA model, the relation among topics has not been depicted, but for real-world applications, there is a common condition that topics have correlations among them. Therefore, a hierarchical topic model emerged to fill the need.

Hierarchical latent Dirichlet allocation (hLDA) (Griffiths and Tenenbaum 2004) is an unsupervised hierarchical topic modeling algorithm that is aimed at learning topic hierarchies from data. In this model, the distributions of topic hierarchies are represented by a process called the nested Chinese restaurant process. Each node in the hierarchy tree is associated with a topic, where a topic is a distribution across words. A document is generated by choosing an *L*-level path from the root to a leaf. Therefore, for each document, the topics are only repeatedly sampled along the same path. Likewise, the Pachinko allocation model (PAM) was proposed in Li and McCallum (2006) for unsupervised hierarchical topic modeling. The difference between hLDA and the PAM is that the correlation of topics in the PAM is a directed acyclic graph (DAG) instead of only a tree in hLDA. Furthermore, the leaves of the DAG in the PAM represent individual words in the vocabulary, whereas each interior node represents the topic, which is a distribution over its children. Therefore, the concept of a topic is extended to distributions not only over words but also over other topics.

On the basis of hLDA and the PAM, several hierarchical topic models were proposed later. Supervised hierarchical latent Dirichlet allocation (SHLDA) (Nguyen et al. 2013) allows documents to have multiple paths through the tree by leveraging information at the sentence level. Hierarchical labeled LDA (HLLDA) (Petinot et al. 2011) is a Bayesian model that introduced a label prior into hLDA. There is also one-to-one correspondence between a label and topic. A semisupervised hierarchical topic model (SSHLLDA) is proposed in Mao et al. (2012) and is aimed at exploring new topics automatically in data space while incorporating information from the observed hierarchical labels into the modeling process. The labeled Pachinko allocation model (LPAM) (Bakalov et al. 2012) can automatically assign keywords to a given taxonomy in multilabel documents. A semisupervised hierarchical model called the Wikipedia-based Pachinko allocation model (WPAM) is proposed in Kataria et al. (2011). It was designed to learn accurate entity disambiguation models from Wikipedia. In reference (Ma et al. 2012), a labeled four-level Pachinko allocation model (L-F-L-PAM) is proposed to capture correlations among multiple labels.

A correlated topic model (CTM) is proposed in Blei and Lafferty (2007). As in the above-mentioned hierarchical topic models, the topics are not independent in the CTM, but only pairwise correlations among topics are modeled by a logistic normal distribution.

In LDA, the topics are fixed for the whole corpus, and the number of topics is assumed to be known. Wang and McCallum (2006) proposed topic over time (TOT) to jointly model both word co-occurrences and localization continuously. In a hierarchical Dirichlet process (HDP) (Teh et al. 2006a), which is a Bayesian nonparametric topic model, the number of topics does not need to be specified in advance and is determined by collection during posterior inference.

### Extension of document attributes

In LDA, both the order and other attributes of documents were not considered. Nonetheless, besides the word occurrence statistics of documents, other document attributes such as author, title, geographic location, and links also provide guidance on “topic” discovery. There were many success stories in this kind of research in recent years.

In the author-topic model (Rosen-Zvi et al. 2004), the generative process is as follows: choose an author at random; generate a word based on the topic probability distribution of this author; repeat the above steps until the document generation is finished. In the relational topic model (Chang and Blei 2010), each document is modeled as in LDA, and the distances between topic proportions of documents reflect the links between documents. The dynamic topic model (Blei and Lafferty 2006) takes into account the ordering of the documents and yields a richer posterior topical structure than LDA does. A Dirichlet-multinomial regression (DMR) topic model (Mimno and McCallum 2012) provides a log-linear prior for document-topic distributions, and its aim is to incorporate arbitrary types of observed document features, such as author and publication venue.

### Extension of word attributes

In the BoW of LDA, the order of words in a document is not considered either. Therefore, a number of extensions of the LDA model have been attempted to eliminate the exchangeability of words. For example, a new topic model proposed by Wallach (2006) relaxes the BoW assumption and assumes that a word is generated by a topic depending on its previous word.

### Other kinds of data

One advantage of LDA is that the document-generative process can be adapted to other kinds of analyses, keeping only the analogy between document-topic-word and other kinds of objects. Therefore, the basis of topic modeling is the appointment of three objects: documents, words, and topics. For example, in computer vision, researchers have drawn a direct analogy between images and documents. The collections of “visual words” make up the images. Thus, visual patterns (topics) can be discovered by topic modeling. This way, topic modeling has been applied, for example, to image classification (Fei–Fei and Perona 2005).

## The toolkits for topic models

With the development of topic models, several toolkits have appeared for the broad application of these topic models. The toolkits below are mainly used in natural language processing.

1. Gensim
  Gensim (Rehurek 2008) is a free Python library that is aimed at automatic extraction of semantic topics from documents. The input of Gensim is a corpus of plain text documents. There are several algorithms in Gensim, including LSI, LDA, and Random Projections to discover semantic topics of documents. Once the semantic topics are discovered, the plain text documents can be queried for topical similarity against other documents.
2. Stanford topic modeling toolbox (TMT)
  Stanford TMT (Ramage and Rosen 2009) was written in the Scala language by the Stanford NLP group. It is designed to help social scientists or other researchers who wish to analyze voluminous textual material. The input of Stanford TMT can be text in Excel or other spreadsheets. There are several algorithms in TMT, including LDA, Labeled LDA, and PLDA.
3. MALLET
  MALLET (McCallum 2002) is a Java-based package for natural language processing, including document classification, clustering, topic modeling, and other text mining applications. There are implementations of LDA, of the PAM, and of HLDA in the MALLET topic modeling toolkit.
4. Other open source software
  Besides the above toolkits, David Blei’s Lab at Columbia University (David is the author of LDA) provides many freely available open-source packages for topic modeling. These open-source packages have been regularly released at GitHub and include the dynamic topic model in C language, a C implementation of variational EM for LDA, an online variational Bayesian for LDA in the Python language, variational inference for collaborative topic models, a C++ implementation of HDP, online inference for HDP in the Python language, a C++ implementation of sLDA, hLDA, and a C implementation of the CTM.

## The use of topic models in bioinformatics

Above all, topic modeling aims to discover and annotate large datasets with latent “topic” information: Each sample piece of data is a mixture of “topics,” where a “topic” consists of a set of “words” that frequently occur together across the samples. This essence of topic modeling strongly accords with biologists’ interests, which include discovering latent patterns in massive biological data. Hence, in recent years, extensive studies have been conducted in the area of biological-data topic modeling. In this section, we discuss existing studies on topic models applied to bioinformatics. First, the process of selection of articles is described.

### Selection of articles

The selection process involves four steps. For example, first, we search for potentially relevant articles published from 1999 to 2016 in PubMed and Web of Science. In PubMed, the search string is (bioinformatics[MeSH Terms] OR computational biology[MeSH Terms]) AND (“topic model” OR “topic modeling”). In Web of Science, the search string is topic: [topic-model OR topic-modeling) AND topic:((biology OR medicine) OR biomedicine]. Second, relevant articles (judging by the title and abstract) are retrieved for more detailed evaluation. Third, we search the bibliographies of relevant articles for additional references. Finally, all the retrieved articles are screened by means of the following inclusion criteria: 1) original research published in English; 2) processing of biological data; and 3) the use of LSI, PLSA, LDA, or other variants of the LDA model. At the same time, we exclude articles that meet the following criterion: the use of a topic model for pure text data. This search strategy identified 30 publications.

To conduct an integrative analysis of these 30 articles, we study them on the basis of three themes: the tasks of a topic model, the type of a topic model, and the analogy between document-topic-word and a biological object. These three themes also form the foundation for deep understanding of the use of topic models in bioinformatics and are discussed next.

### The tasks in a topic model for bioinformatics

**Fig. 5:**
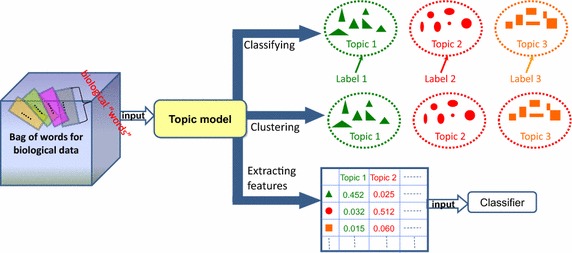
The tasks of a topic model in bioinformatics

First of all, we place special emphasis on the roles and tasks of a topic model in bioinformatics. By exploring the relevant studies, we found that the tasks of a topic model for biological data are mainly focused on three concepts: biological data clustering analysis, biological data classification, and biological data feature extraction. To illustrate the relation among these three tasks, a diagram is shown in Fig. 5 (The tasks of a topic model in bioinformatics). The triangles, circles, and rectangles of different colors represent biological samples processed by a topic model. Their color differences indicate that these biological samples have high probability for different topics. In other words, they can be clustered or classified to different topics. The three concepts will be discussed individually in the following sections.

#### The use in biological data clustering analysis

As discussed in “Topic modeling” section the learning process of an LDA model is completely unsupervised; hence, its research area is currently concentrated on unlabeled data. The major function of a topic model is clustering of documents in a text domain: each document is represented by a topic probability distribution, and the documents that have high probability for the same topic can be considered a cluster. Hence, unlike in traditional clustering, a topic model allows data to come from a mixture of clusters rather than from a single cluster. Naturally, data clustering is also the major function of topic modeling for biological data, but the “topic” has a special biological meaning.

As shown in Fig. 5, several biological “topics” were extracted from a BoW of biological data by topic model clustering, and can also be regarded as biological “clusters.” It is important to note that clustering analysis is intended for unlabeled data. Hence, topic model clustering can only discover topics but not automatically return the corresponding biological labels. In this section, several examples of related articles will illustrate this kind of research, which predominates in the use of biological-data topic modeling. These studies are described in groups based on the type of biological data and are displayed in chronological order.

First, many studies have been conducted on the topic modeling of expression microarray data. In these studies, gene-sample in this dataset was likened to word-document, and each sample was modeled as a mixture over latent topics. Rogers et al. (2005) and Masada et al. (2009) utilized latent process decomposition (LPD) for discovery of group structure across samples and genes. Because the description of expression microarray data is a matrix of real numbers instead of a non-negative integer matrix, LPD introduced Gaussian distributions to LDA in place of word multinomial distributions. Thereafter, a PLSA model was employed by Bicego et al. (2010a) for extraction of biclusters; this model simultaneously groups genes and samples.

For protein interaction data, Sinkkonen et al. (2008) proposed an infinite topic model to find functional gene modules (topics) combined with gene expression data. In the process of topic discovery, this topic model focuses on the relation among documents; hence, it is also suitable for clustering of other relational data.

To query experiments relevant to particular biological questions, Caldas et al. (2009) applied LDA to experimental genomic data. A query corresponds to one microarray, and the search result is a set of the most similar microarrays. In the BoW of that work, one word type corresponded to one gene set; consequently, the count of differentially expressed genes in gene sets was regarded as equivalent to the count of words. Put another way, the query was encoded as a vector containing the number of differentially expressed genes. Then, each experiment corresponded to a document, which contained a mixture of the components (topics), and each component (topic) corresponded to a distribution over the gene sets. Finally, on the basis of the discovered components, those authors used the principles of text queries for an experimental query.

Given a large collection of fluorescent images, Coelho et al. (2010) utilized LDA to identify the subcellular localization patterns in these images. Their work is similar to what is done in computer vision: an image is represented by mixtures of multiple fundamental patterns (topics), and the key points are defined as visual words.

For gene sequence data, the desirable task is to characterize a set of common genomic features shared by the same species. Chen et al. (2010, 2012a, b) analyzed the genome-level composition of DNA sequences by means of LDA. First, they represented the DNA sequences by N-mer frequencies. After that, genome sequences were assumed to be documents, and the N-mers were regarded as “words.” Next, the genome-level statistical patterns (topics) were discovered by introducing an LDA model. Each inferred topic represented a certain component of the whole genome.

The study by Chen et al. (2011) was focused on abundance data from microbial-community taxa, including protein-coding sequences and their NCBI taxonomical levels. In that study, the LDA model with background distribution (LDA-B) extends the LDA model by adding a background distribution of commonly shared functional elements. The LDA-B model was used to discover functional groups: the genome set served as the document corpus, which contained a mixture of functional groups; each functional group (topic) was a weighted mixture of functional elements; the functional elements served as “words.”

For genome annotation data, Konietzny et al. (2011) employed LDA to directly identify functional modules of protein families. First, to process genome annotations as documents, a fixed-size vocabulary of words was defined on the basis of annotations, and one word could be considered a functional descriptor. Then, the topics inferred by LDA represent functional modules.

Liu et al. (2011) and Zhang et al. (2012a) described a topic model that can discover functional microRNA regulatory modules (FMRMs) in expression profiles of microRNAs and mRNAs. In that study, they mapped topics to functional modules, samples to documents, and the samples were profiled with a set of microRNAs and a set of mRNAs. Consequently, the functional modules inferred by modified correspondence latent Dirichlet allocation (Corr-LDA) acted as a bridge between microRNAs and mRNAs. The Corr-LDA has been successfully used to annotate images by caption words. Finally, an expression dataset from a murine experimental model was emulated by this topic model for research on human breast cancer.

In order to analyze cellular endpoints from in vitro high-content screening (HCS) assays, Bisgin et al. (2013) also introduced LDA. For each drug, they generated a document for each of the four time points. The document was assumed to contain occurrences of endpoint measurements (words). They supposed that the expression of the HCS endpoints can be modeled as a probability distribution of “topics.” Next, the probabilistic associations between topics and drugs were built by LDA.

#### The use for biological-data classification

Besides the clustering for unlabeled biological data, a topic model can accomplish classification tasks for labeled biological data. In other words, a topic model can not only discover topics but also make the topics match the true biological labels. Nonetheless, as unsupervised learning models, PLSA and LDA offer no obvious way of incorporating a supervised set into their learning procedure. Therefore, for these types of studies, the LDA model needs to be adapted one way or another.

As shown in Fig. 5, like clustering, a topic model classifies discoveries of biological “topics” from a BoW of biological data. Meanwhile, these biological “topics” are labeled with true biological terms, which can also be called labels. For labeled data, this mode of operation of a topic model is beneficial for interpretation of a topic and provides tools for tuning the generated topics to match an end-use application. Moreover, compared with other classification approaches such as support vector machine (SVM) (Rubin et al. 2011), the classification result of a topic model under certain conditions shows competitive performance. Similarly, several examples of relevant articles will illustrate this kind of projects in this section.

First, for expression microarray data, the research subject of studies by Perina et al. (2010) is similar to that in Rogers et al. (2005) and Pratanwanich and Lio (2014): there is also a straightforward analogy between the pairs word-document and gene-sample. Nonetheless, Perina et al. introduced biologically aware latent Dirichlet allocation (BaLDA) to perform a classification task that extends the LDA model by integrating document dependences and starts from the LPD. BaLDA does not contain the assumption present in both PLSA and LDA that each gene is independently generated given its corresponding latent topic. A priori knowledge about relations among genes is expressed in terms of gene categorization. In the training phase, this categorization (topic) can be computed beforehand; in the testing phase, it can also be estimated. Finally, the authors demonstrated the usefulness of BaLDA in two classification experiments. Another study on classification of gene expression data is a pathway-based LDA model proposed by Pratanwanich and Lio (2014). That study was aimed at learning drug-pathway-gene relations by treating known gene-pathway associations as prior knowledge. In that study, they drew an analogy between drug-pathway-gene and document-topic-word. They regarded genes as words and viewed a pathway as a topic. First, pseudo drug documents were produced in the training phase, and the model was learned by parameter inference. Then, for a new pseudo drug document, this model can predict responsiveness of the pathway to a new drug treatment.

For patient-related texts constructed from clinical and multidimensional genomic measurements, Dawson and Kendziorski (2012) proposed a survival-supervised latent Dirichlet allocation (survLDA) model, which is a supervised topic model. The survLDA model was inspired by sLDA (Mcauliffe and Blei 2008) applied to evaluation of movies, and addressed the following tasks: characterization of cancer subtypes and classification of individual patients according to those subtypes. They consider each patient’s text a “document,” and “words” describe clinical events, treatment protocols, and genomic information from multiple sources. Then, “topics” are the implicit categories of patients.

At last, in the problem of genomic sequence classification, La Rosa et al. (2015) consider genomic sequences to be documents, small fragments of a DNA string of size *k* to be words, and the topics discovered by LDA are assigned taxonomic labels. It is noteworthy that this study is similar to research in Chen et al. (2010, 2012a, b). Nonetheless, the difference is that the topic discovered in data on genomic sequences not only has a probability distribution over words but also corresponds to a true taxonomic label.

#### The use for extraction of biological data features

In topic modeling, the term “space of documents” has been transformed into “topic” space, and the “topic” space is smaller than word space. Therefore, a probabilistic topic model is also a popular method of dimensionality reduction for collections of text documents or images. Likewise, the dimensionality reduction is a common and often necessary task in biological-data analysis. As shown in Fig. 5, we can utilize a topic model to project the original feature space of biological data onto the latent topic space. After the reduction of dimensionality in this way, other algorithms such as a classifier can process the resulting topic features at a later step, as in common feature space.

One study in this field was carried out for magnetic resonance imaging (MRI). To improve the classification accuracy of discrimination between normal subjects and patients with schizophrenia, Castellani et al. (2010) applied the PLSA model: each image was regarded as a document, the shape descriptors of images served as visual words, and then the geometric patterns of the brain surface were considered visual topics. They extracted a generative score from the learned model, which was used as input of an SVM for the classification task.

For protein sequence data, Pan et al. (2010) proposed a hierarchical latent Dirichlet allocation-random forest (LDA-RF) model to predict human protein–protein interactions. First, the local sequence feature space was projected onto latent semantic space (topics) by an LDA model; this topic space reflects the hidden structures between proteins and is the input of the next step. Then, the probability of interaction of two proteins was predicted by a random forest model based on the topic space.

Just as in other studies on expression microarray data (Rogers et al. 2005; Masada et al. 2009), Bicego et al. (2010b, 2012) also drew an analogy between the pairs word-document and gene-sample. Nonetheless, the latter study introduced the PLSA, LDA, and LPD models into the microarray classification task. In the training phase, a topic model was employed to extract a feature vector, which is actually a set of topics. Then, a classifier based on the K-Nearest Neighbor rule was trained in the transformed training set. In the testing phase, the same feature extraction process was applied to the test set, which was then classified using the trained classifier.

Zhang et al. (2015) used a topic model for assigning metagenomic reads to different species or taxonomical classes. First, they represented each metagenomic read (document) as a set of “k-mers” (words). Then, the LDA model was applied to the reads and generated a number of hidden “topics.” Finally, they used SKWIC—a variant of the classical K-means algorithm—to cluster these reads represented by topic distributions.

#### Uses in other bioinformatics domains

In addition to the above studies, there are several projects where a topic model was applied to biological data in an innovative way. It is hard to find out the basic laws of this field because of its diversity. Nonetheless, examples of relevant articles are presented below.

To use a topic model for bimolecular annotations, Masseroli et al. (2012), Pinoli et al. (2013, 2014) defined a co-occurrence matrix as the annotations. In the matrix, if a gene is annotated with an ontological term, then the value is 1.0; otherwise, it is 0. Given an annotation corpus represented by this matrix, they used the modified topic model to estimate the term probability distributions over a topic and the topic probability distributions over genes. Then, they were able to rebuild the annotation matrix. An element of this matrix gives an estimate of the probability of a gene annotated to a term. It should be noted that although both the above study and the study in Konietzny et al. (2011) are about genome annotation data, Pinoli and coworkers used a topic model as a matrix decomposition tool rather than a clustering algorithm.

### Topic models applied to bioinformatics

From the description of the relevant articles above, we can deduce that most of the studies on topic modeling in biological data have utilized existing topic models directly, such as PLSA and LDA. Both PLSA and LDA are relatively simple topic models and serve as the basis for other, extended topic models. Meanwhile, the basic assumption in LDA or PLSA may be violated in a special application scenario; then, the generative process and inference algorithm need to be readjusted. Hence, some investigators in recent years tried to improve the LDA model for new biological contexts. The types of topic models that were used in the 30 above-mentioned articles are summarized in Table 3.

**Table 3:** A summary of topic model types in the relevant studies (see “Topic models applied to bioinformatics” section)

References	Types of topic model
Castellani et al. (2010), Bicego et al. (2010a, b, 2012), Masseroli et al. (2012), Pinoli et al. (2013)	PLSA
Caldas et al. (2009), Chen et al. (2010, 2012a, b), Coelho et al. (2010), Pan et al. (2010), Bicego et al. (2010b), Konietzny et al. (2011), Zhang et al. (2012a), Bisgin et al. (2013), Lee et al. (2014), Pinoli et al. (2014), Pratanwanich and Lio (2014), Randhave and Sonkamble (2014), Youngs et al. (2014), La Rosa et al. (2015), Zhang et al. (2015)	LDA
Rogers et al. (2005), Masada et al. (2009)	LPD
Liu et al. (2011)	Corr-LDA
Sinkkonen et al. (2008)	topic model for relational data
Perina et al. (2010)	BaLDA
Dawson and Kendziorski (2012)	survLDA
Fang et al. (2015)	Semi-parametric transelliptical topic model
Chen et al. (2011)	LDA-B

### “Document-word-topic” in biological data

In the above introduction to topic models, we can see that the gist of topic modeling is appointment of three objects: documents, words, and topics. Similarly, the descriptions of the relevant studies above also indicate that the key task of topic modeling in biological data is drawing an appropriate analogy between document-topic-word and a biological object. Table 4 groups the above studies by the analogy between terms used in text mining and those in biology.

**Table 4:** A summary of the analogies between document-topic-word and a biological object in the relevant studies (see ““Document-word-topic” in biological data” section)

Reference	Words	Topics	Documents	Biological dataset
Rogers et al. (2005), Masada et al. (2009), Perina et al. (2010), Bicego et al. (2010a, b, 2012), Lee et al. (2014)	Genes	Functional groups	Samples	Expression microarray data
Masseroli et al. (2012), Pinoli et al. (2013, 2014), Youngs et al. (2014)	Ontological terms	Latent relationship	Proteins	Protein annotations
Chen et al. (2010, 2012a, b), La Rosa et al. (2015), Zhang et al. (2015)	K-mers of DNA sequences	Taxonomic category/components of the whole genome	DNA sequences	Genomic sequences
Caldas et al. (2009)	Gene sets	Biological process	Experiments	Gene expression dataset
Coelho et al. (2010)	Object classes	Fundamental patterns	Images	Fluorescence images
Konietzny et al. (2011)	A fixed-sized vocabulary of words based on the gene annotations	Functional modules of protein families	Genome annotations	A set of genome annotations
Bisgin et al. (2013)	Endpoint measurements	Diagnostic topics	Drugs	Expression of the HCS endpoints
Chen et al. (2011), Randhave and Sonkamble (2014)	Functional elements (NCBI taxonomic level indicators, indicator of gene orthologous groups and KEGG pathway indicators)	Functional groups	Samples	Genome set
Pan et al. (2010)	Local sequential features	Latent topic features	Protein sequences	Protein–protein interaction dataset
Castellani et al. (2010)	Shape descriptors	Brain surface geometric patterns	Images	Magnetic resonance images
Pratanwanich and Lio (2014)	Genes	Pathways	Gene expression profiles	Gene expression data
Dawson and Kendziorski (2012)	Clinical events, treatment protocols, and genomic information from multiple sources	The category of patients	Patients	Patient’s text constructed from clinical and multidimensional genomic analyses

As shown in the above summary, no matter what kind of biological data is modeled, the basic idea is that a biological dataset resembles a set of documents. That is, the dataset consists of mixtures of biological processes, which can be thought of as topics, and a biological process consists of a set of biological words, which can be likened to the words used to present a topic.

## The trends in applications of topic models to bioinformatics

Overall, most of the studies where a topic model is applied to bioinformatics are task oriented; relatively few studies are focused on extensions of a topic model. It is obvious, however, that relaxing the basic assumption of LDA or PLSA is a desirable approach because of the availability of many other a priori pieces of information, such as documents’ interactions, the order of words, and knowledge on the biology domain. Likewise, there are many scenarios that require violation of the basic assumption of topic models, for example, protein–protein interaction. This kind of study on improvement of models is urgently needed. In addition, there is significant motivation to reduce the time taken to learn topic models for very large biological data. For this purpose, the respective advantages of classic inference algorithms such as complexity and accuracy may be combined into some new accelerated algorithms (Porteous et al. 2008), such as “real-time” topic modeling that has been proposed in Yao et al. (2009), Hoffman et al. (2010). In short, the existing topic models still leave a lot to be desired for application to bioinformatics.

Aside from several possible research projects mentioned above, after in-depth analysis of the relevant studies, two promising and worthwhile research projects are proposed in this paper.

### Predicting protein function via a hierarchical multilabel topic model

With the rapid accumulation of proteomic and genomic datasets, computational methods for automated annotation of protein functions are in high demand. The problem of protein function prediction is a typical multilabel classification task whose solutions are protein functional annotations. For protein function prediction, a multilabel topic model can emulate the protein as a document and the function label as a topic. This method can not only obtain the function probability distributions of protein instances but also directly provide the word probability distributions over functions. Nonetheless, several key problems also need to be addressed. First of all, the number of function labels is large. For example, the number of gene ontology (GO) terms is greater than 19,600. If the correspondence between the topics and the GO terms is one to one, then the number of topics may be much greater than the number of words. This condition will yield infinite perplexity in the protein function dataset. Second, as opposed to PLSA or LDA, the function labels of a protein are no longer independent. For example, GO terms are organized as a hierarchical structure such as a DAG in GO, and the number of hierarchies is 15. Therefore, for a protein with hierarchical labels, researchers must consider how to utilize the hierarchical relation between labels to find the corresponding topic for each label. All in all, predicting a protein function by means of a hierarchical multilabel topic model is a challenging and worthwhile task.

### Visualization of biological topics and user interfaces

Topic models provide new exploratory structure for big biological data: the topics are displayed as the most frequent words (as shown in Fig. 2). By contrast, topics that are assigned a biological label will make the results easier to understand for a biologist. Therefore, how to display a topic with a specific biological meaning is the key task of the practical use of a topic model.

Overall, for biologists, easy-to-understand visualization of the discovered topics in a user interface is essential for topic modeling. Exploration of an effective interface to biological data and its inferred topic structure are a long-term undertaking.

## Conclusion

The above studies showed that a topic model can accomplish the task of clustering and classification of biological data. Furthermore, each topic is interpreted as a probability distribution over words. That is, compared with black-box algorithms, a topic model can produce a more understandable result and thus may help a biologist to interpret the finding. Meanwhile, unlike traditional clustering, a topic model allows data to come from a mixture of clusters rather than from a single cluster. These characteristics may be useful in bioinformatics.

The studies on application of topic models to bioinformatics are only beginning, and further research on improvement of models will soon become an urgent necessity, especially in bioinformatics. We believe that topic models are a promising method with numerous applications to biomedical research.

## Abbreviations

LSI: latent semantic indexing
PLSA: probabilistic latent semantic analysis
LDA: latent Dirichlet allocation
BoW: bag of words
EM: expectation maximization algorithm
EP: expectation-propagation
VB: variational Bayesian inference
sLDA: supervised LDA
discLDA: discriminative variation on LDA
medLDA: maximum entropy discrimination LDA
LLDA: labeled LDA
PLLDA: partially labeled LDA
HDP: hierarchical Dirichlet process
hLDA: hierarchal latent Dirichlet allocation
PAM: Pachinko allocation model
DAG: directed acyclic graph
SHLDA: supervised hierarchical latent Dirichlet allocation
HLLDA: hierarchical labeled LDA
SSHLLDA: semisupervised hierarchical topic model
LPAM: labeled Pachinko allocation model
WPAM: Wikipedia-based Pachinko allocation model
L-F-L-PAM: labeled four-level Pachinko allocation model
CTM: correlated topic model
TOT: topic over time
TMT: Stanford topic modeling toolbox
LPD: latent process decomposition
LDA-B: LDA model with background distribution
FMRMs: functional microRNA regulatory modules
Corr-LDA: correspondence latent Dirichlet allocation
HCS: high-content screening
SVM: support vector machine
BaLDA: biologically aware latent Dirichlet allocation
survLDA: survival-supervised latent Dirichlet allocation
MRI: magnetic resonance imaging
LDA-RF: latent Dirichlet allocation-random forest
GO: gene ontology
